# Risk factors associated with fatality of severe fever with thrombocytopenia syndrome: a meta-analysis

**DOI:** 10.18632/oncotarget.19163

**Published:** 2017-07-11

**Authors:** Yuxin Chen, Bei Jia, Yong Liu, Rui Huang, Junhao Chen, Chao Wu

**Affiliations:** ^1^ Department of Laboratory Medicine, Nanjing Drum Tower Hospital, Nanjing University Medical School, Nanjing 210008, Jiangsu Province, China; ^2^ Department of Infectious Disease, Nanjing Drum Tower Hospital Clinical College of Nanjing Medical University, Nanjing, 210008, Jiangsu Province, China; ^3^ Department of Experimental Medicine, Nanjing Drum Tower Hospital, Nanjing University Medical School, Nanjing, 210008, Jiangsu Province, China

**Keywords:** severe fever with thrombocytopenia syndrome, risk analysis, meta-analysis

## Abstract

Severe fever with thrombocytopenia syndrome is an emerging life-threatening infectious disease identified in 2009. Given high case-fatality rate among patients with severe fever with thrombocytopenia syndrome, identification of the risk factors at acute phase associated with fatality is crucial for treatment. Therefore, we aimed to meta-analytically evaluate risk factors of fatal clinical outcome of severe fever with thrombocytopenia syndrome. 238 fatal cases and 873 non-fatal cases from 12 studies were included in this meta-analysis. Elder age and high viral load were significantly associated with fatal clinical outcome. Further, severe fever with thrombocytopenia syndrome patients with fatal clinical outcome had significantly reduced level of albumin and platelet count, higher level of serum alanine aminotransferase, aspirate aminotransferase, lactic acid dehydrogenase and creatinine phosphokinase, and prolonged activated partial thromboplastin time, comparing with mild patients. These disturbed parameters function as predictors to warn fatal clinical outcome of the disease. Moreover, ribavirin has a minimal impact to alleviate disease progression of severe fever with thrombocytopenia syndrome. In conclusion, our finding demonstrates a panel of factors associated with fatality of SFTS disease, which have important implications during clinical practice.

## INTRODUCTION

High case fatality rate and wide epidemic of severe fever with thrombocytopenia syndrome (SFTS) has been a serious public health burden. This emerging virus infection, first identified in China in 2009, caused by a novel bunyavirus belonging to genus *Phlebovirus*, family *Bunyaviridae* [1]. The outbreak of SFTS has been identified in at least 20 provinces of China. Besides, SFTS-like patients were also reported in USA, Japan and Korea, but none of them had oversea travel history, suggesting worldwide distribution of SFTS virus [1–3]. SFTS disease was, most likely, acquired by being bitten via ticks such as *Haemaphysalis longicornis* [4] or direct contact with infected animal tissues [5]. Cases of person-to-person transmission were also noted [6–9]. The epidemic analysis showed the seroprevalence of SFTS is 0.84–6.37% among Chinese population residing in the endemic areas [10]. Given the 12–30% of case fatality rate among SFTS patients [11, 12], identification of the risk factors associated with fatal clinical outcome of SFTS at early stage of the disease is crucial for SFTS treatment. Further, whether current anti-viral drug, ribavirin, is effective for SFTS disease remains controversial. Therefore, it is urgent to collectively analyze the factors associated with fatal outcome of SFTS disease and evaluate the efficacy of current anti-viral treatment with real-world data.

The symptoms of SFTS infections are variable, ranging from an acute self-limited febrile illness to life threatening. The typical clinical presentation is characterized by sudden onset of fever, fatigue, gastrointestinal symptoms, leukopenia, and thrombocytopenia. Based on the clinical progression of SFTS, the natural history of SFTS is divided into three distinct stages: fever, multiorgan dysfunction syndrome (MODS), and convalescence. The fever phase of illness (day 0–6) was defined as early acute phase of infection, while the second stage of disease (day 7–13) could further turn into MODS, a major reason result in fatality. Most SFTS patients were recovered fully within 2–3 weeks since abrupt fever. However, severe cases of SFTS further proceed to the stage of multiple organ dysfunction (MOD) and disseminated intravascular coagulation (DIC), and the majority of severe cases lead to fatality within 7–14 days since illness onset. A number of published studies had described the potential risk factors contributing to fatality of SFTS disease, including demographic characteristics and a panel of clinical laboratory tests upon during early acute stage of SFTS disease. However, each prospective cohort included a relative small number of SFTS patients majorly from single medical center. Further, various clinical parameters were analyzed in each study, which reached inconsistent conclusions. In this present meta-analysis, we attempted to draw a precise conclusion based on the published literature. The predictors identified in the current study could be applied to warn fatal clinical outcome of SFTS.

## RESULTS

### Study selection

**Figure 1:**
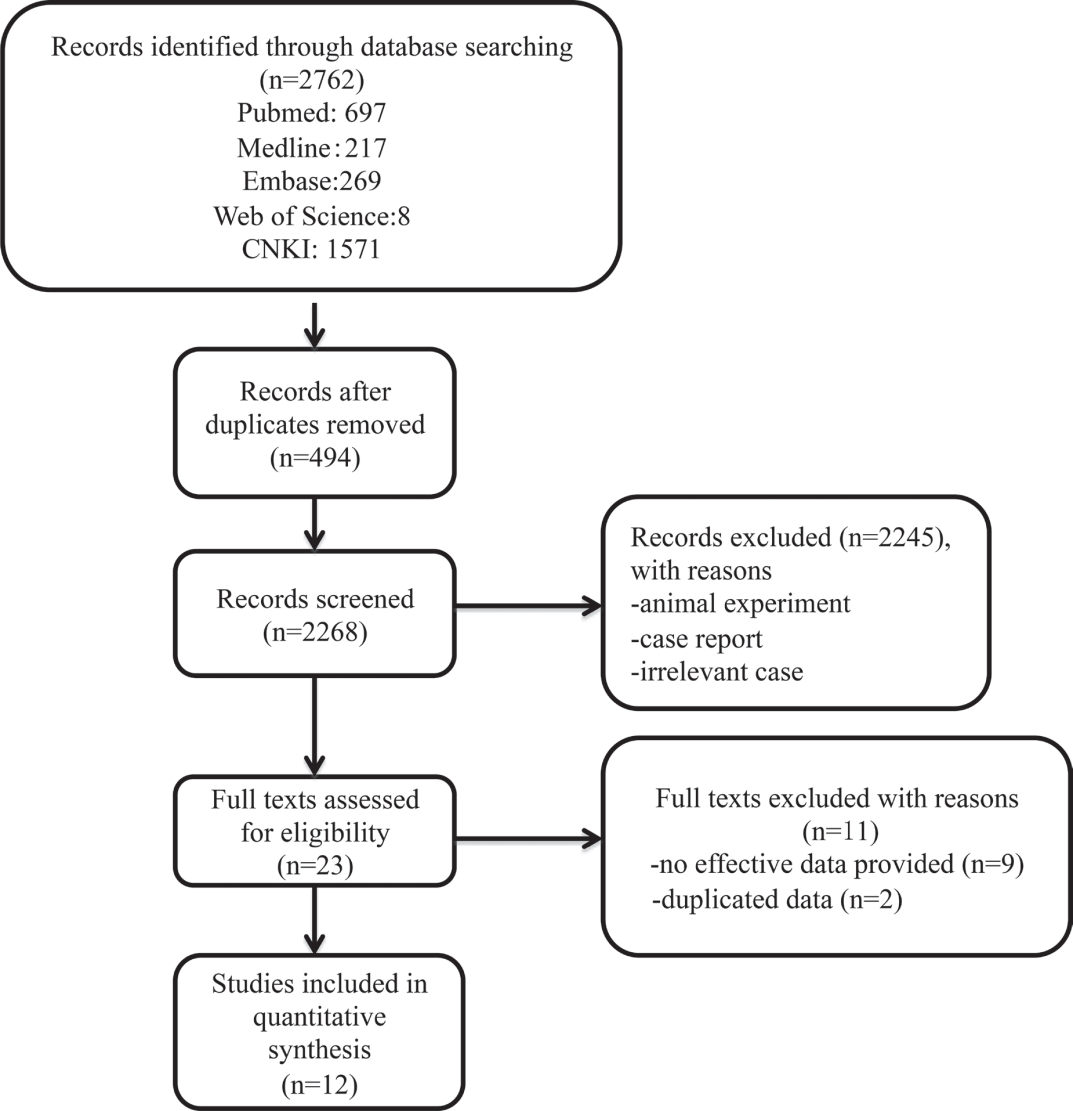
A Flow diagram showing the selection of studies

The flow chart of literature search on risk factors analysis of fatal clinical outcome of SFTS was shown in Figure 1. We retrieved 2762 records electronically. After 494 duplicates and 2245 irrelevant studies were removed, studies that met the following criteria were included after preliminary screening: (1) the patients were diagnosed of SFTS infection by reverse-transcriptase PCR (RT-PCR), (2) the clinical outcome of their patients were categorized by “fatal” versus “non-fatal’. A total of 23 articles were evaluated for eligibility. After checking details, 9 studies were excluded due to lack of effective data. Among them, 2 studies only had the clinical information between severe and non-severe SFTS patients [13, 14]. Further, we noticed that 4 studies [1, 15–17] are updated analyses that included the data from previous published study [13, 14, 18, 19], respectively. Under close scrutiny, the study made by Shin J *et al.* [1] had more detailed clinical parameters (including AST, LDH, CK, interval days, neutrophil, APTT, and ribavirin treatment) for SFTS patients, while Choi *et al.* [18] did not recorded detailed information. Xiong *et al.* [19] recorded detailed data including LDH, age, PLT, and lymphocyte count, while such data was lacking in the study from Zhang *et al.* [17]. Ultimately, 12 full articles were eligible for meta-analysis [1, 3, 15, 17–25]. 11 were published in English, and 1 study published in Chinese. Details of full text screening and study selection process were illustrated in Figure 1.

### Study characterization and quality assessment

The detailed information extracted from included studies was shown in Table 1. All were prospective studies conducted in Asia. 1 study was in Western Japan from 2010–2014, 2 studies were from South Korea during 2013–2015, and the rest of 9 studies were from China. Among the studies from China, 5 studies were conducted in the Hubei Provinces during the year of 2010 and 2012–2015, 1 studies were conducted in Shandong province based on the admitted SFTS patientsduring 2011, 1 studies were in Henan Province from 2011–2013, 2 studies were in Jiangsu Province from 2010 to 2014. Current studies included in this meta-analysis have covered the majority of provinces with high incidence of SFTS. There is no significant difference in terms of average age and gender. All studies included in this meta-analysis were considered to be of high quality (Table 2).

**Table 1:** Studies and characteristics of SFTS patients included in the meta-analysis

Study ID	Published year	Year of admitted SFTS patients	Region	Fatal cases (n)	Non-fatal Cases (n)	Median age	Male/Female
Zhang Y et al. [23]	2012	2010	Hubei, China	8	41	54.4	NA
Gai Z et al. [15]	2012	2011	Shandong, China	11	48	61.5	31/28
Sun L et al. [22]	2014	2012	Hubei, China	3	28	NA	19/12
Cui N et al. [20]	2014	2011–2013	Henan, China	54	303	61	202/155
Li J et al. [21]	2014	2011–2013	Jiangsu, China	3	21	NA	13/11
Shin J et al. [2]	2015	2013	South Korea	16	19	69	17/18
Peng C et al. [24]	2016	2014	Hubei, China	9	44	53.8	30/23
Zhao H et al. [25]	2016	2010–2014	Jiangsu, China	7	33	57.6	19/21
Kato H et al. [4]	2016	2013–2014	Western Japan	15	34	78	17/32
Choi SJ et al. [18]	2016	2013–2015	South Korea	46	74	69	61/59
Xiong et al. [19]	2016	2015	Hubei, China	34	145	58	71/108
Zhang et al [17]	2017	2015	Hubei, China	21	94	60	45/70

**Table 2:** Assessment of the quality of 12 included studies

Study ID	Published year	Clear definition of study population?	Clear Definition of outcomes and outcome assessment ?	Independent assessment of outcome parameters?	Sufficient duration of follow-up?	No selective loss during follow-up?	Important confounders and prognostic factor identified?
Zhang Y et al. [23]	2012	Yes	Yes	No	Yes	Yes	Yes
Gai Z et al. [15]	2012	Yes	Yes	No	Yes	Yes	Yes
Sun L et al. [22]	2014	Yes	Yes	No	Yes	Yes	No
Cui N et al. [20]	2014	Yes	Yes	Yes	Yes	Yes	Yes
Li J et al. [21]	2014	Yes	Yes	No	Yes	Yes	Yes
Shin J et al. [2]	2015	Yes	Yes	Yes	Yes	Yes	Yes
Peng C et al. [24]	2016	Yes	Yes	No	Yes	Yes	Yes
Zhao H et al. [25]	2016	Yes	Yes	No	Yes	Yes	Yes
Kato H et al. [4]	2016	Yes	Yes	No	Yes	Yes	Yes
Choi SJ et al. [18]	2016	Yes	Yes	Yes	Yes	Yes	Yes
Xiong et al. [19]	2016	Yes	Yes	Yes	Yes	Yes	Yes
Zhang et al. [17]	2017	Yes	Yes	Yes	Yes	Yes	Yes

### Epidemiological factors that influence the SFTS disease progression

**Figure 2:**
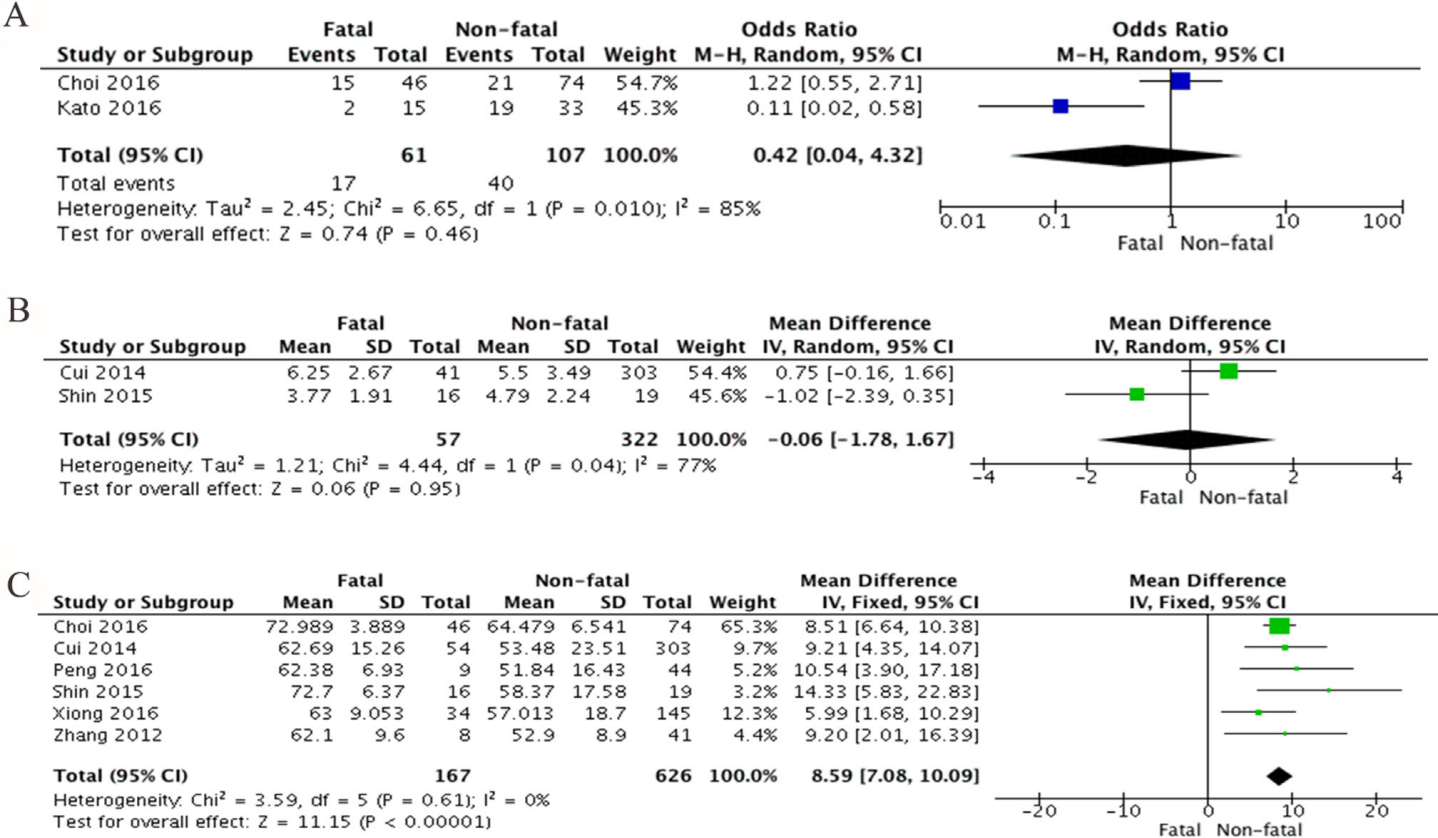
Forest plots of meta-analysis on a panel of demographic factors and fatal outcome of SFTS disease, including (**A**) tick bite expressed as odds ratio (OR), (**B**) interval days between illness onset to hospitalization expressed as mean difference in days, and (**C**) age of SFTS patients expressed as mean difference in years.

SFTS disease could be acquired through various transmission modes, including tick bite, contact with infected livestock or infectious blood from SFTS patients. Interestingly, for a major portion of patients, the history of tick bite or the trace of tick bite was confirmed during physical examination. However, the impact of transmission mode on the disease progression remains unclear. 2 studies were analyzed, which includes 61 fatal cases and 107 non-fatal cases. Overall, there is no significant association between tick bite and fatality of SFTS disease (OR = 0.42, 95% CI 0.04 to 4.32, *P* = 0.46) (Figure 2A).

Early admission to hospital and prompt treatment for suspected SFTSV infection is considered to be critical. Data from 2 studies were collected, in which 322 non-fatal cases and 57 fatal cases were included. However, this was no difference in fatal and survived SFTS cases regarding the interval days between onset and admission (MD = –0.06, 95 % CI –1.78 to 1.67, *P* = 0.95) (Figure 2B).

Age is a critical risk factor for fatal outcome of SFTSV infection [1, 13, 20, 23, 24, 26], while one study by Deng *et al.* reached a different conclusion [14]. In current meta-analysis, a total of 6 studies including 626 non-fatal SFTS cases and 167 fatal SFTS cases were evaluated. Deceased patients were significantly more senior compared to those with non-fatal SFTS (MD = 8.59, 95% CI 7.08 to 10.09; I^2^ = 0%; *P* < 0.00001*)* (Figure 2C). No significant heterogeneity was found in this analysis (I^2^ = 0% and *P* = 0.61).

### Clinical laboratory parameters that associated with fatality of SFTS disease

**Figure 3:**
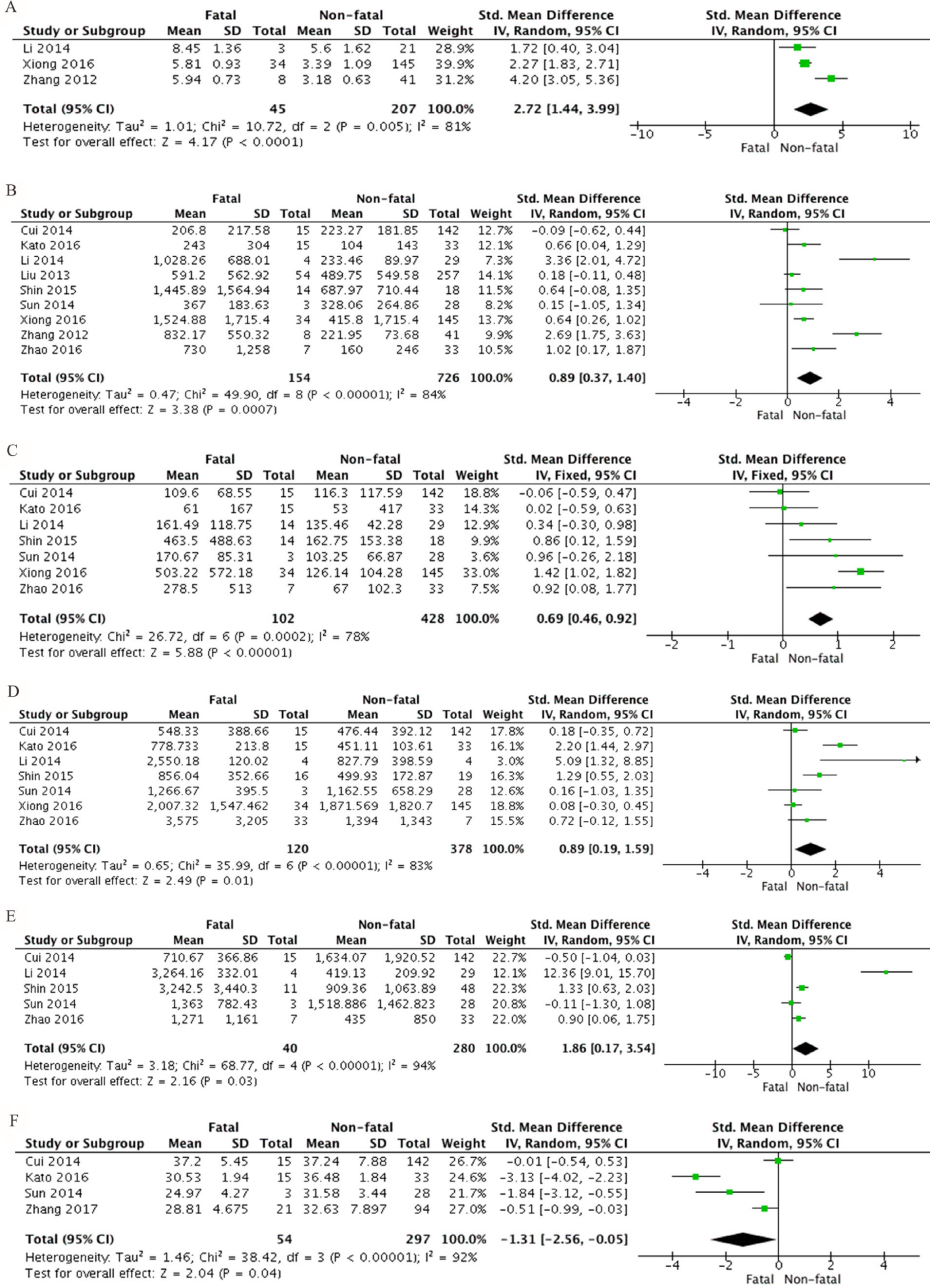
Forest plots of meta-analysis on a panel of clinical key biochemical parameters and fatal SFTS disease, including (**A**) viral load, (**B**) aspirate aminotransferase (AST), (**C**) alanine aminotransferase (ALT), (**D**) lactic acid dehydrogenase (LDH), (**E**) creatinine phosphokinase (CK) and (**F**) albumin (ALB), expressed as standard mean differences.

The clinical laboratory parameters at acute phase were analyzed. Viral load of SFTS bunyavirus was considered to negatively correlate with PLT, but positively correlate with serum enzymes, pro-inflammatory and anti-inflammatory cytokines [21]. High level of viral load was associated with fatal SFTS disease, revealed by our meta-analysis (SMD 2.72, 95% CI 1.44 to 3.99; *P* < 0.0001). (Figure 3A). A substantial heterogeneity was noticed with I^2^ = 81% and *P* = 0.005. Clinical biochemical laboratory analysis showed that, compared to non-fatal SFTS patients, fatal SFTS patients had significantly elevated level of AST (SMD 0.89, 95% CI 0.37 to 1.40, *P* = 0.0007), ALT (SMD 0.69, 95% CI 0.46 to 0.92, *P* < 0.00001), LDH (SMD 0.89, 95% CI 0.19 to 1.59, *P* = 0.01), CK (SMD 1.86, 95% CI 0.17 to 3.54, *P* = 0.03), but reduced albumin (SMD –1.31 95% CI –2.56 to –0.05, *P* = 0.04) (Figure 3B–3F). Obvious heterogeneities were observed from this group of meta-analysis (AST: I^2^ = 84% and *P* < 0.00001; ALT: I^2^ =78% and *P* = 0.0002; LDH: I^2^ = 83% and *P* < 0.00001; CK: I^2^ = 94% and *P* < 0.00001; ALB: I^2^ = 92% and *P* < 0.00001).

**Figure 4:**
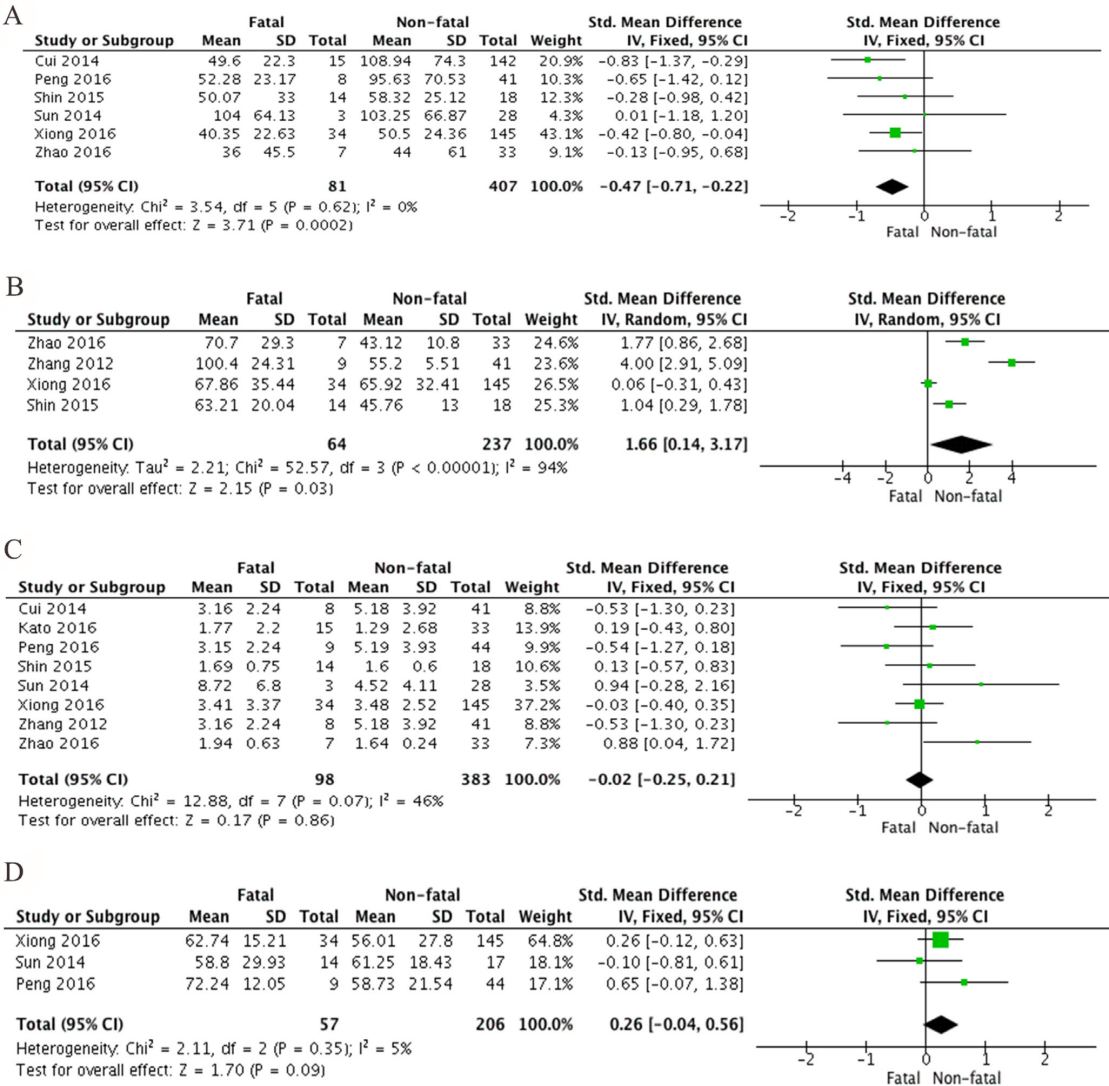
Forest plots of meta-analysis on a panel of hematologic parameters and the fatality of SFTS disease, including (**A**) platelet count, (**B**) activated partial thromboplastin time (APTT), (**C**) leukocyte counts and (**D**) percentage of neutrophils, expressed as standard mean differences.

Further, hematology tests showed that the level of PLT was also significantly diminished in fatal SFTS patients when comparing non-fatal SFTS patients (SMD –0.47, 95% CI –0.71 to –0.22, *P* = 0.0002). There was no heterogeneity detected from this analysis (I^2^ = 0%, *P* = 0.62) (Figure 4A). APTT is also significantly prolonged in fatal SFTS patients compared to non-fatal (SMD 1.66, 95% CI 0.14 to 3.17, *P* = 0.03). There was a significant heterogeneity derived, and I^2^ is 94% with a *P* value < 0.00001 (Figure 4B). Further, both leukocytes and neutrophils were not significantly different between fatal and non-fatal SFTS patients (SMD 0.02, 95% CI –0.02 to 0.21, *P* = 0.86 and SMD 0.26, 95% CI –0.04 to 0.56, *P* = 0.09). A moderate heterogeneity was revealed from meta analysis of leukocyte (I^2^ = 46%, *P* = 0.07), and a modest heterogeneity was demonstrated from neutrophil analysis (I^2^ = 5%, *P* = 0.35) (Figure 4C–4D).

### Effect of Ribavirin for SFTS treatment

**Figure 5:**
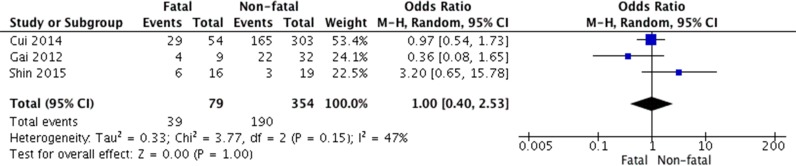
Forest plot of the association between administration of ribavirin and survival of SFTS disease

Ribavirin was extensively used for treating SFTS patients; however, its efficacy to suppress SFTSV replication is highly concerned (25). 79 fatal cases and 354 survived cases from 3 studies were included. Our meta-analysis indicated that its effectiveness to influence SFTS disease progression is minimal (OR 1.00, 95% CI 0.40 to 2.53, *P* = 1.00). There was no heterogeneity detected from this analysis (I^2^ = 47% and *P* = 0.15) (Figure 5).

### Sensitivity analysis and publication bias

There are several meta-analyses with significant heterogeneity. However, meta-regression analysis is only applied for the meta-analysis containing more than 10 studies. Here a panel of sensitivity analyses was performed to evaluate the influence of each individual study. Pooled SMD were not significantly affected by any individual study (Supplementary Figures 1–4).

**Figure 6:**
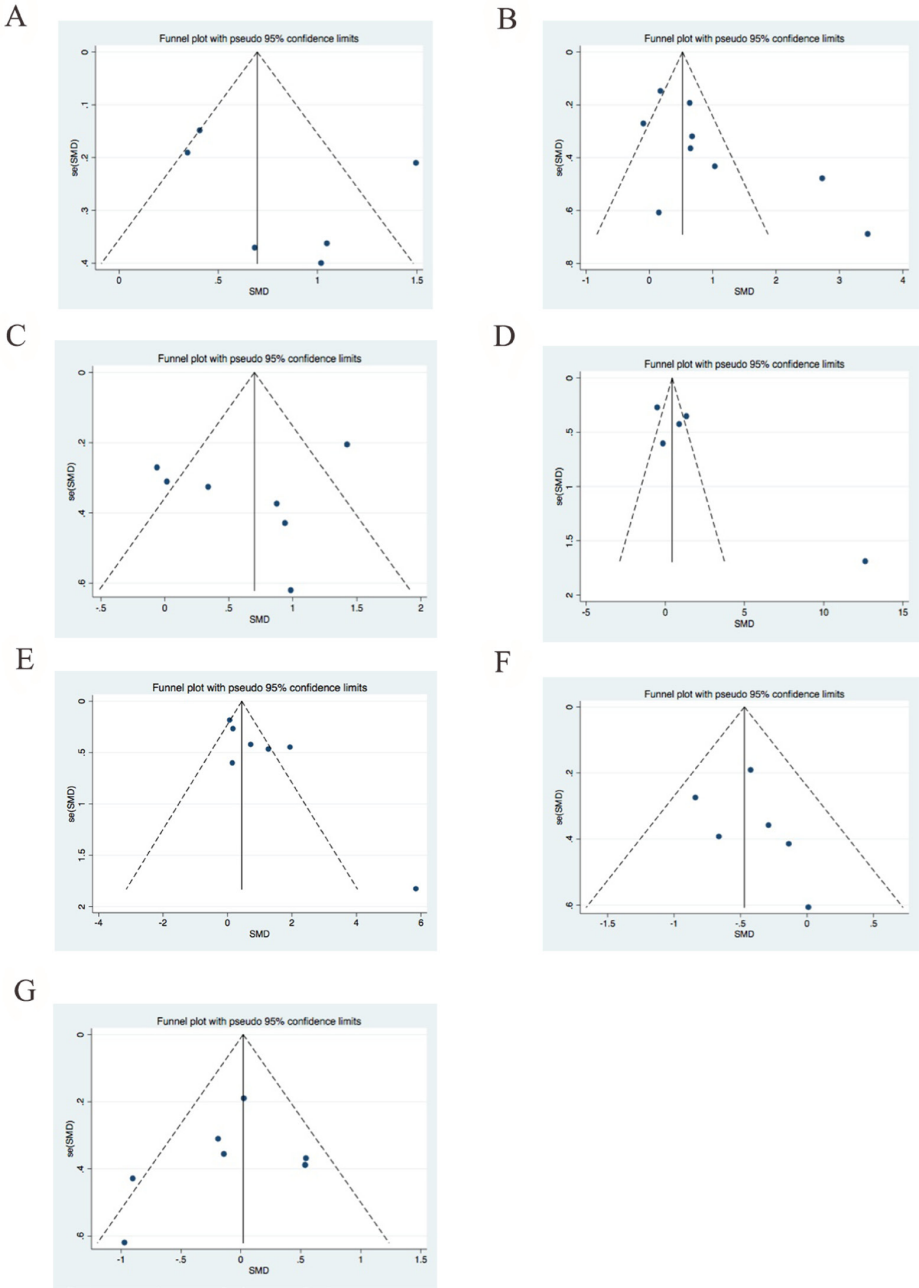
Funnel plots for publication bias for (**A**) age, (**B**) AST, (**C**) ALT, (**D**) CK, (**E**) LDH, (**F**) PLT, (**G**) leukocyte count among fatal and non-fatal SFTS disease.

Further, funnel plots and Egger’s tests were conducted to evaluate the publication bias of meta-analyses for which include 5 or more than 5 studies. There is no significant asymmetry identified from funnel plots for age, AST, ALT, CK, LDH, PLT and leukocytes, and *Pr >* | z | value for the Begg’s test were 0.34, 0.095, 0.453, 0.327, 0.536, 0.188 and 0.602, respectively (Figure 6A–6G). Egger’s test showed consistent results with Begg’s test (data not shown).

## DISCUSSION

For an emerging infectious disease, characterization of clinical features, identification of risk factors associated with disease progression, and evaluation of therapy efficacy is essential to facilitate diagnosis and treatment in clinical practice. Risk factors contributing to fatal clinical outcome of SFTS patients were sparsely investigated, and, unfortunately, no consistent conclusion derived. Therefore, we performed this comprehensive meta-analysis to identify the potential risk factors that might predict the fatality of SFTS disease.

Epidemiology analysis has revealed that all age groups were susceptible to SFTS infection, but only senior people infected with SFTS bunyavirus suffered from severe clinical presentations and even died of SFTSV infection [26]. Indeed, in our meta-analysis we showed that patients with elder age were associated with the fatality of SFTS disease, possibly due to their low immunity.

The role of transmission mode in determining the status of SFTS progression remains unclear. The major transmission modes include tick bite, contact of livestock [10] and human-to-human transmission via infectious blood or body fluid [8]. It has been reported that human-to-human transmission of SFTS via blood leads to mild presentations [9], suggesting specific transmission mode might influence the disease progression. However, from our analysis, the transmission mode of tick bite does not correlate with fatal clinical outcome. In contrast, viral load at early phase is highly associated with fatality of SFTS disease in our analysis.

A panel of clinical biochemical and homological laboratory parameters were also systematically evaluated. First, pronounced level of hepatic and myocardial enzymes, including ALT, AST, LDH and CK, were observed in the group of deceased SFTS patient, indicating possible injuries of liver, heart and other organs in fatal SFTS patients. This could be a consequence of viral replication within the specific tissue or an indirect outcome of cell death through inflammatory cytokine storm [13, 16]. Further, coagulation disorders featured by diminished number of platelet and extended APTT were significantly correlated with poor prognosis of SFTS disease. Indeed, it has been shown that in a mouse infection model, platelets were adhered to SFTSV, which further promote the clearance of splenic macrophages [27]. In addition to thrombocytopenia, leukopenia is the typical hallmark of patients with SFTS. However, our meta-analysis showed that the number of leukocytes and neutrophils were not significantly different between groups. It could be derived from individual variety of basal level of leukocyte and neutrophil cells. Change of leukocytes from basal level to illness onset might be more valuable to predict the prognosis of SFTS disease. These laboratory parameters mentioned above were routinely measured, therefore, yielding a highly predictive value to distinguish the patients at higher risk of fatality, who should get additional attention during treatment. Interestingly, although 12 studies were from 3 different Asian countries, 2 Korean studies [3, 18] and 1 Japanese study [5] did not show any potential heterogeneity, demonstrated by our sensitivity analysis and publication bias analysis, suggesting risk factors identified in this study for fatality of SFTS disease were reliable and consistent among different countries.

There are several additional risk factors reported, including inflammatory cytokines (IL-6 and IL-10) [13, 21], chemokines (IL-8, monocyte chemotactic protein 1 and macrophage inflammatory protein 1b) [23], immune cells (NK cell, myeloid dendritic cells and monocyte percentage) [19, 22, 24]. Unfortunately, only limited studies measured these immune parameters; therefore, they were not included in our current meta-analysis. It will be interesting to determine the correlation of these immune parameters and clinical outcome of SFTS disease in the context of larger sample size. Besides, specific clinical presentations (abdominal pain, neurological disorder and respiratory symptoms) were also reported as potential risk factors. However, the clinical symptom is a subjective evaluation during clinical practice, which is difficult for future application of predictors for outcome of SFTS disease. Of note, an epidemiology study suggested that platelet derived growth factor-B polymorphism was correlated with risk of SFTS in Chinese individuals, demonstrating that host genetic variations may influence susceptibility to SFTS disease and potentially clinical outcome of SFTS disease [28].

Currently, the major treatment for SFTS disease is synthetic nucleoside antiviral agent ribavirin and other general supportive therapy. Ribavirin was shown to have inhibitory activity against both DNA and RNA viruses, including Crimean-Congo hemorrhagic fever, hemorrhagic fever with renal syndrome, hantavirus pulmonary syndrome, and rift valley fever [29–32]. Although ribavirin is effective for SFTS bunyavirus *in vitro* [33], ribavirin treatment could not improve clinical laboratory parameters, e.g., platelet counts [34]. The efficacy of ribavirin should be further tested in small animal model with SFTS infection [35]. Our analysis also showed that ribavirin treatment did not reduce the risk of fatal clinical outcome of SFTS disease. Interestingly, late admission to hospital is not a risk factor to influence SFTS disease progression, further confirming current treatment might not effective to reduce the case fatality ratio. Therefore, a novel anti-viral agent such as a potent neutralizing antibody is essential to reduce the severity and fatality of SFTS disease. More importantly, an effective vaccine against SFTS disease is urgently needed.

## MATERIALS AND METHODS

This meta-analysis was performed and written according to the PRISMA (Preferred Reporting Items for Systematic Reviews and Meta Analyses) statement as a guideline (http://www.prisma-statement.org/).

### Search strategy and inclusion criteria

On Jan 1th, 2017, we carefully conducted a systematic search of PubMed, Medline, Embase, and Web of Science databases, and Chinese National Knowledge Infrastructure (CNKI) database searches were conducted for all eligible papers (published between Nov 1, 2008 and Jan 15, 2017; English and Chinese publication) using the search terms “severe fever with thrombocytopenia syndrome”. We also included additional studies manually searched from references of the original articles and reviews.

The inclusion criteria were as follows: (1) the article reported a prospective study and had been accepted for publication with full text available; (2) all cases were given a diagnosis of SFTS infection by reverse-transcriptase PCR (RT-PCR), the fatal and non-fatal clinical outcome was evaluated, and possible risk factors were reported.

### Quality assessment and data extraction

The quality of 12 observational studies was systematically evaluated. Two blinded reviewers (Y.C. and B.J.) independently analyzed each studies included in this meta-analysis according to a critical review checklist of the Dutch Cochrane Centre [34]. The key points of this checklist are as follows: 1) clear definition of study population; 2) clear definition of outcomes and outcome assessment; 3) independent assessment of outcome parameters; 4) sufficient duration of follow-up; 5) no selective loss during follow-up; and 6) important confounders and prognostic factors identified.

### Data extraction

For our meta-analysis, we extracted the following predefined variables: authors, year of sample collection, age, and the size of each group. The focus of our analysis was to evaluate the potential factors that link with the fatal clinical outcome of SFTS infection. The factors included in our meta-analysis were presence of tick bite, the interval between onset and admission, the age of patient, viral load, aspartate aminotransferase (AST), alanine transaminase (ALT), albumin (ALB), lactate dehydrogenase (LDH), creatine kinase (CK), platelet count (PLT), activated partial thromboplastin time (APTT), leukocyte and neutrophils along with the corresponding 95% CI. All these parameters were collected during the first stage of SFTS disease (day 0–6). We also evaluate whether using ribavirin improve the clinical outcome of SFTS.

### Statistical analysis

The statistical analyses were carried out using Review Manager (RevMan version 5.2.5; Nordic Cochrane Centre, Copenhagen, Denmark) and STATA (version 12.0; StataCorp, College Station, Texas, USA) software. If the published data only showed medians and interquartile ranges rather than means and SDs, the means and SDs were calculated as described by Hozo et al. [35]. As the lab parameters might be measured in different methods and reported across different labs, the absolute values of these parameters were further converted into common unit by calculating standardized effect size. Heterogeneity among studies was checked using the chi-squared test and I^2^ statistics. I^2^ test was conducted to measure the proportion of the overall variation that attributable to between-study heterogeneity, ranging from 0% to 100%, > 50% is considered as evidence of heterogeneity. If *P* < 0.05 or I^2^ > 50%, a random effect model was used. In contrast, a fixed effect model was used when *P* ≥ 0.05 and I^2^ ≤ 50%. In addition, we conducted a sensitivity analysis to investigate the potential source of heterogeneity and assess the strength of our finding by sequentially excluding one study. Furthermore, publication bias among the studies was investigated by using Begg’s funnel plot and the Egger’s test.

## SUPPLEMENTARY MATERIALS FIGURES


