# Supplementary material for: Risk factors associated with fatality of severe fever with thrombocytopenia syndrome: a meta-analysis
Source: Oncotarget. 2017 Jul 11;8(51):89119–29. doi: 10.18632/oncotarget.19163 (PMC5687674; doi:10.18632/oncotarget.19163)
Supplement: Supplementary file 1 [file oncotarget-08-89119-s001.pdf]

Risk factors associated with fatality of severe fever with thrombocytopenia syndrome: a meta-analysis

SUPPLEMENTARY MATERIALS

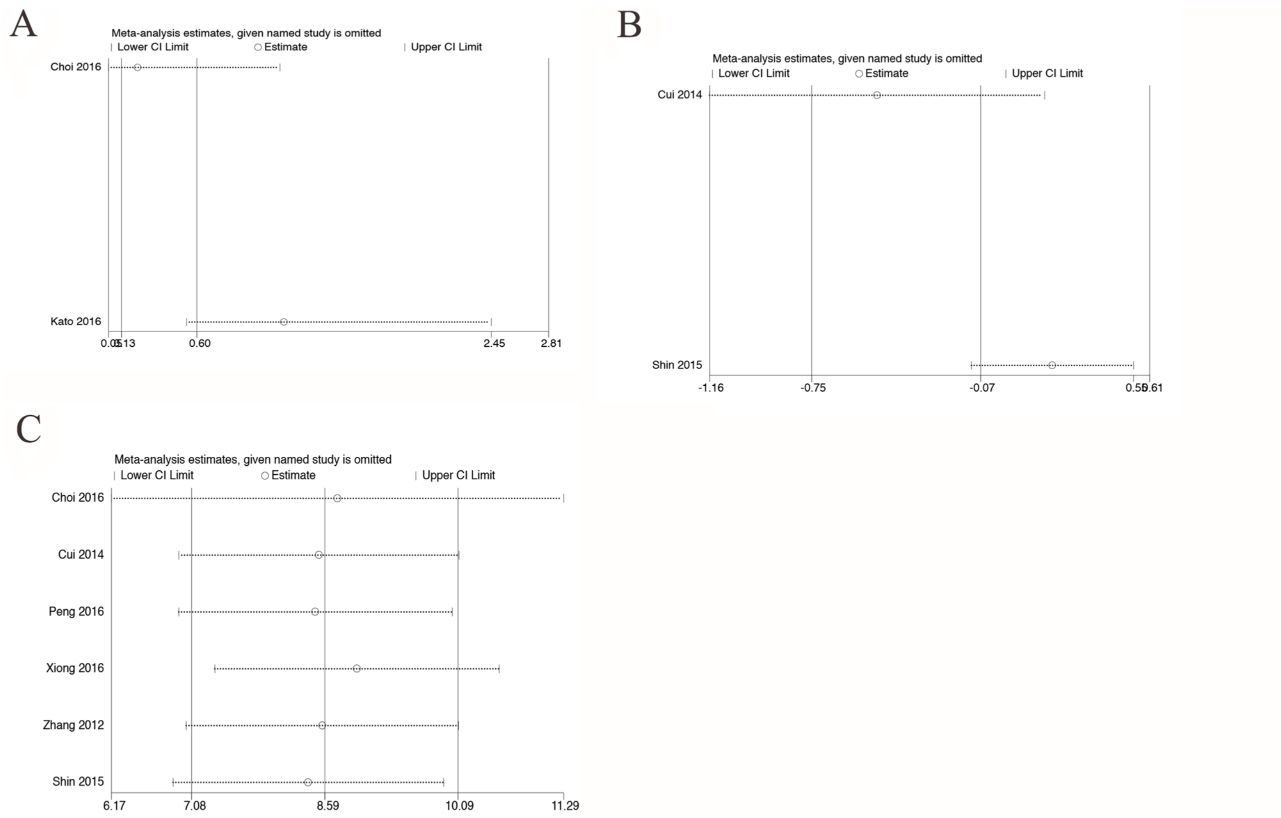

Supplementary Figure 1: Sensitivity analysis of the association between (**A**) tick bite, (**B**) interval days between illness onset to hospitalization, (**C**) age and fatality of SFTS disease.

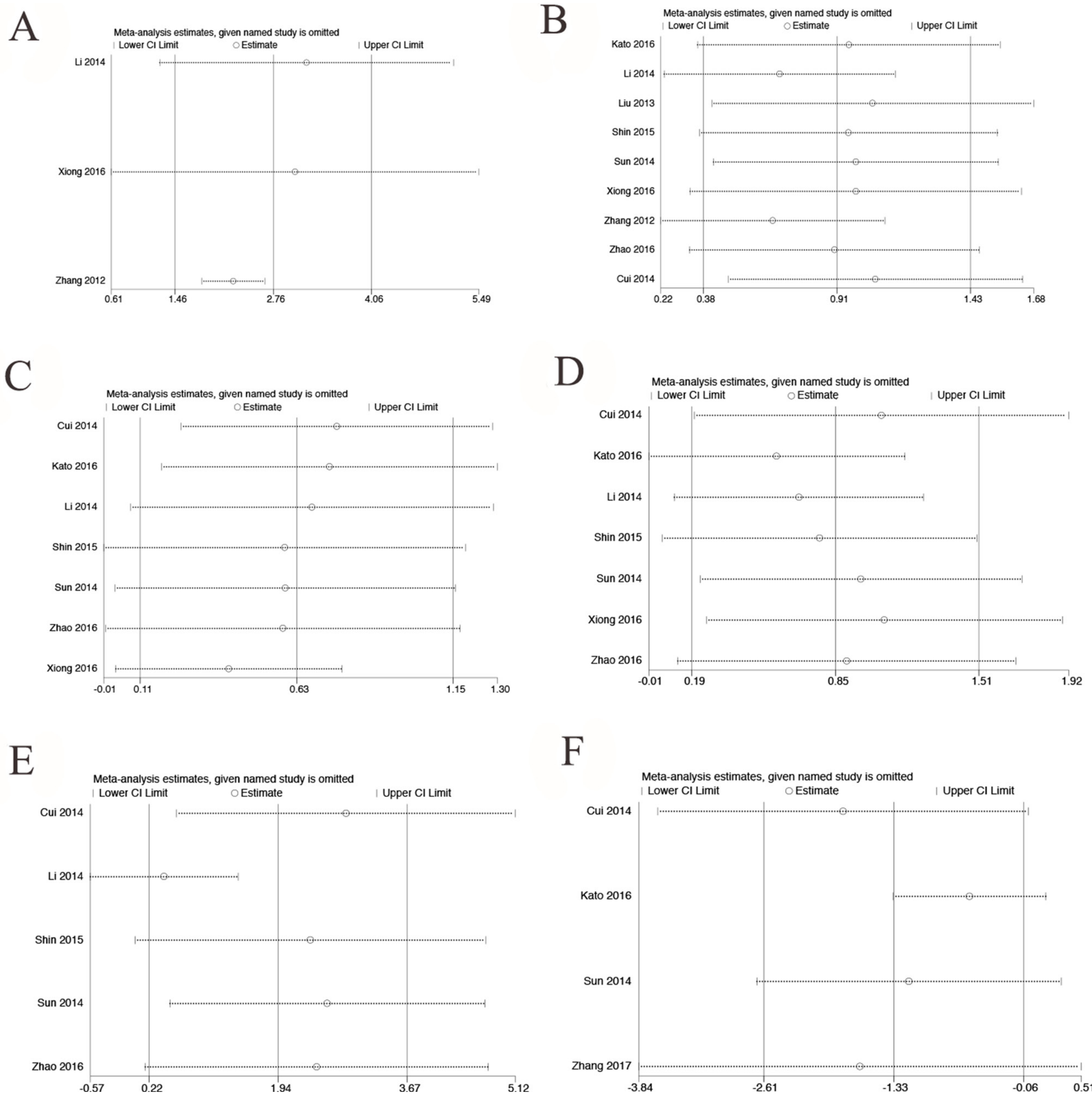

Supplementary Figure 2: Sensitivity analysis of the association between (**A**) viral loads, (**B**) AST, (**C**) ALT, (**D**) LDH, (**E**) CK as well as (**F**) ALB and fatality of SFTS disease.

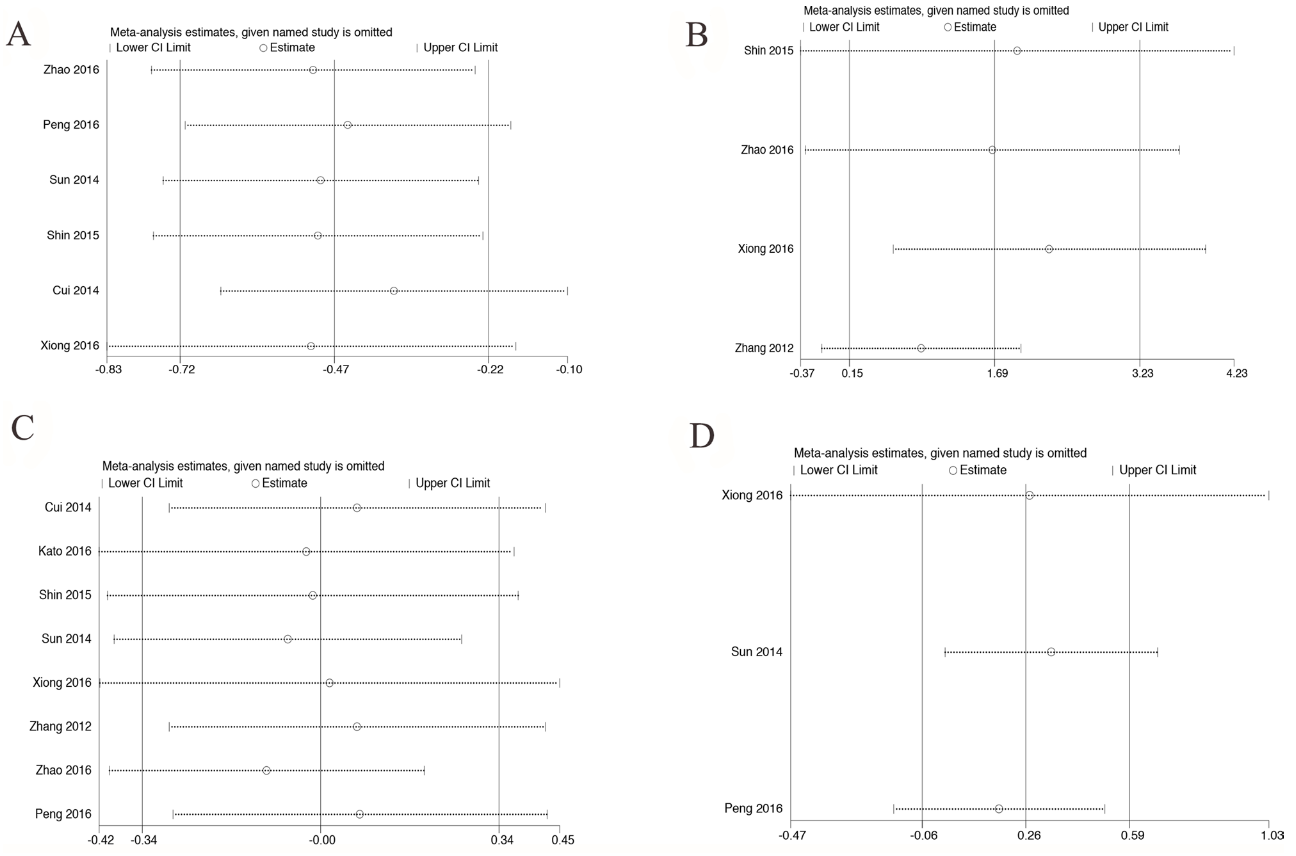

Supplementary Figure 3: Sensitivity analysis of the association between (**A**) PLT, (**B**) APTT, (**C**) lymphocytes, (**D**) neutrophils and fatality of SFTS disease.

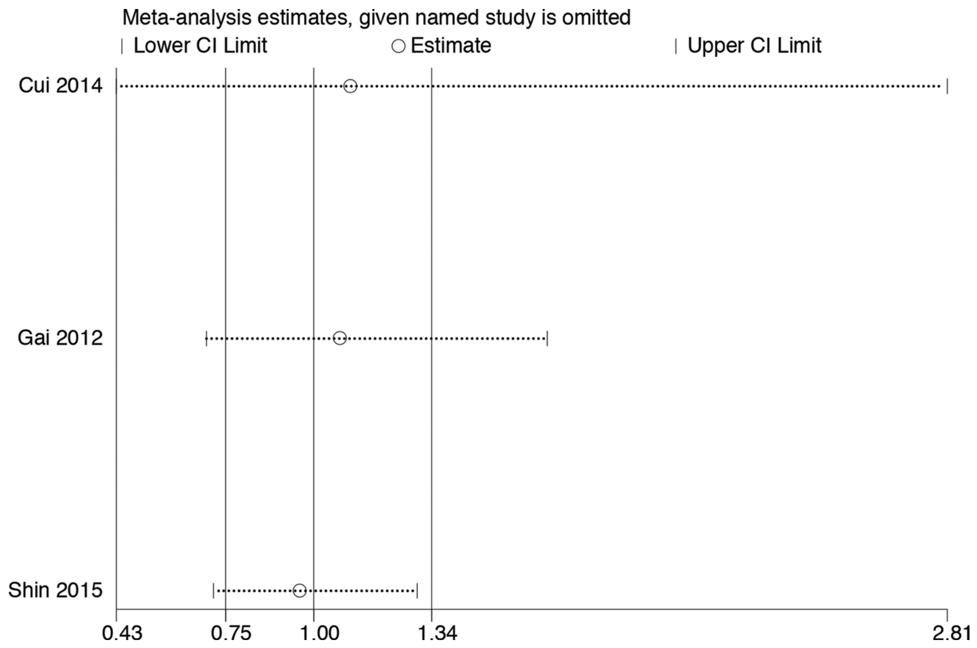

Supplementary Figure 4: Sensitivity analysis of the association between administration of ribavirin and survival of SFTS disease.
